# Humoral response after a fourth dose of SARS-CoV-2 vaccine in immunocompromised patients. Results of a systematic review

**DOI:** 10.3389/fpubh.2023.1108546

**Published:** 2023-03-23

**Authors:** Silvia Martinelli, Domenico Pascucci, Patrizia Laurenti

**Affiliations:** ^1^Department of Life Sciences and Public Health, Università Cattolica del Sacro Cuore, Rome, Italy; ^2^Department of Woman and Child Health and Public Health, Fondazione Policlinico Universitario Agostino Gemelli IRCCS, Rome, Italy

**Keywords:** immunocompromised, fourth dose, immune system, second booster, COVID-19, SARS-CoV-2

## Abstract

**Background and objective:**

The fourth dose the COVID-19 vaccine was first proposed to immunocompromised patients. The aim of the article is to systematically review the literature and report the humoral response and outcomes after the fourth dose administration in people with impaired immune system.

**Methods:**

Published studies on the humoral response, efficacy and safety of the fourth dose of the COVID-19 vaccine were analyzed in various settings of immunocompromised patients. We conducted systematic searches of PubMed, Cochrane Library and WHO COVID-19 Research Database for series published through January 31, 2023, using the search terms “fourth dose” or “second booster” or “4th dose” and “Coronavirus” or “COVID-19” or “SARS-CoV-2.” All articles were selected according to the PRISMA guidelines.

**Results:**

A total of 24 articles including 2,838 patients were comprised in the systematic review. All the studies involved immunocompromised patients, including solid organ transplant recipients, patients with autoimmune rheumatic disease, patients with human immunodeficiency virus (HIV) and patients with blood cancers or diseases. Almost all patients received BNT162b2 or mRNA-1273 as fourth dose. All the studies demonstrated the increase of antibody titers after the fourth dose, both in patients who had a serological strong response and in those who had a weak response after the third dose. No serious adverse events after the 4th dose have been reported by 13 studies. COVID-19 infection after the fourth dose ranged from 0 to 21%.

**Conclusion:**

The present review highlights the importance of the fourth dose of covid-19 vaccines for immunocompromised patients. Across the included studies, a fourth dose was associated with improved seroconversion and antibody titer levels. In particular, a fourth dose was associated with increasing immunogenicity in organ transplant recipients and patients with hematological cancers, with a very low rate of serious side effects.

## Introduction

The infectious disease caused by the novel Coronavirus SARS-CoV-2 (COVID-19) has been deemed one of the most critical global health emergencies in recent years and vaccine development has become crucial for limiting disease transmission, especially in fragile people and patients with impaired immune system (1). Worldwide, more than 5 billion people have undergone at least one dose of the COVID-19 vaccine and ~ 4.9 billions were fully vaccinated according to World Health Organization (WHO) (2). In Europe, the percentage of people who received a booster dose is 30.9% (2). In the USA, a third dose of COVID-19 vaccine has been administered to ~ 33% of the population) (3).

The European Center for Disease Prevention and Control and European Medicine Agency recommend the administration of the fourth dose to people above 60 as well as vulnerable persons of any age, administered at least 4 months after the previous one, with a focus on people who have received a previous booster more than 6 months ago (4). In March 2022, the U.S. Food and Drug Administration allowed a fourth dose for immunocompromised people and anyone 50 years of age or older (5).

On the other hand, in Israel, administration of the fourth dose started from January 2022 for workers in health service and people over 60 years of age (6–8). Currently, a fourth dose has been granted for Israelis in immunocompromised groups.

Immunocompromised people represent ~3% of the overall population, and deserve particular attention because of possible suppression or over-activation of the immune system attributable to the primary disease or concurrent treatment (9). In this group, SARS-CoV-2 infection and viral shedding is more severe and persistent, and the risk of death is higher (10). Given the reduced immune responses, immunodeficient patients are less prone to develop serious complications of COVID-19 and cytokine storm. However, they are more likely to develop opportunistic infections that can mimic the symptoms of SARS-CoV-2 infection (11). Therefore, a fourth dose has been proposed for immunocompromised patients, including organ transplant recipients (12–14), people on active treatment for solid tumor, people with hematologic malignancies, patients treated with chimeric antigen receptor (CAR)-T-cell therapy or hematopoietic stem cell transplant, patients with moderate or severe primary immunodeficiency (e.g., common variable immunodeficiency disease, severe combined immunodeficiency, DiGeorge syndrome, Wiskott-Aldrich syndrome), with advanced or untreated human immunodeficiency virus (HIV) infection (people with HIV and CD4 cell counts < 200/mm^3^, history of an AIDS-defining illness without immune reconstitution, or clinical manifestations of symptomatic HIV), on active treatment with high-dose corticosteroids (i.e., 20 or more mg of prednisone or equivalent per day when administered for 2 or more weeks), alkylating agents, antimetabolites, transplant-related immunosuppressive drugs, cancer chemotherapeutic agents classified as severely immunosuppressive, tumor necrosis factor (TNF) blockers, and other biologic agents that are immunosuppressive or immunomodulatory (15).

To date, no systematic reviews have been performed on the immunogenicity of a fourth dose of COVID-19 vaccines in immunocompromised cohorts. The aim of the article is to systematically review the literature and report the current use of the fourth dose in immunocompromised people, the categories of involved patients, and the results obtained till now.

## Methods

### Study design

This is a systematic review of literature that was completed in accordance with Preferred Reporting Project for Systematic Evaluation and Meta-Analysis (PRISMA) guidelines (16, 17).

### Literature search strategy

A literature search for the studies published up to January 31, 2023 was conducted. No restrictions on language or period of publications were applied. Three different electronic databases (Medline, Cochrane Library and WHO COVID-19 Research Database, which also includes Embase, medRxiv and Scopus articles about COVID-19) were searched employing the keywords “COVID-19” OR “coronavirus” OR “SARS-CoV-2” AND “fourth dose” OR “4th dose” OR “second booster”. Other relevant studies found in the references were also retrieved. The Boolean operator “AND” was used to combine parts of the subject terms and “OR” was used to expand the search. To increase the validity data, we removed non-peer-reviewed articles in the preprint database. Only the more informative publications would be chosen when there were similar studies carried out by the same authors and/or institutions.

### Screening of articles for eligibility and data extraction

The articles identified from the databases and additional resources were screened for eligibility. First, the title and abstract were screened. The following inclusion criteria were used: (1) studies including men or non-pregnant women aged 18 and above, who had impaired immune system at the time of vaccination; (2) fourth dose of COVID-19 vaccination as the intervention measure; (3) randomized trials, observational studies, case series or retrospective studies including at least three patients. Studies were limited to human participants and of any follow-up duration and time points. The definition of immunocompromised patients was borrowed by the National Cancer Institute, identifying them as people with “reduced ability to fight infections and other diseases,… caused by certain diseases or conditions, such as AIDS, cancer, diabetes, malnutrition, and certain genetic disorders, … or by certain medicines or treatments, such as anticancer drugs, radiation therapy, and stem cell or organ transplant (18).”

Second, eligible studies that met the next circumstances were rejected: (1) medical news, popular science articles, non-medical papers, reviews, editorials, comments, basic research, conference abstracts; (2) in case of overlapping studies, the less informative was excluded.

Full articles were retrieved and read in the event of any doubt or uncertainty regarding the content relevance during the abstract screening. After a comprehensive list of abstracts was obtained, the articles were retrieved and reviewed in full text.

Two researchers (SM and DP) independently screened all studies and the results were collected and reviewed by a third researcher (PL). In the event of disagreement involving the study selection, the three reviewers collegially discussed to reach a consensus (PL). Two researchers (SM and DP) extracted data according to a predetermined proforma in Microsoft Excel Version 16.45. All key extracted data were reviewed and quality checked at the end of the data extraction phase by two researchers (SM and PL).

Data on study characteristics comprised setting, study design, sample size, dropout and non-response rates, and inclusion and exclusion criteria. Participant data comprised age, sex, and disease and treatment history, reason of impaired immune system or type of immunocompromising disease and immunosuppressive regimen. Intervention related data included vaccine type and brand, dosing schedule, number of participants receiving each type and brand of vaccine, and median or mean interval between doses. Outcome related data comprised assay type, antibody measured, method of measurement, intervals of sample collection, and number of measurements.

### Data synthesis and quality assessment

Data retrieved was studied then synthesized using a descriptive method. The Risk of Bias In Non-randomized Studies of Interventions (ROBINS-I) tool was used to rate risk of bias for non-randomized included studies (19). This tool assesses seven domains: risk of bias from confounding, selection of participants, classification of interventions, deviations from intended interventions, missing data, measurement of outcomes, and selection of the reported results. The Cochrane Risk of Bias 2.0 tool was used for randomized trials (20). The tool is structured into five domains through which bias might be introduced into the result. These were identified based on both empirical evidence and theoretical considerations. Because the domains cover all types of bias that can affect results of randomized trials, each is mandatory, and no further domains should be added. The five domains for individually randomized trials (including cross-over trials) are: bias arising from the randomization process; bias due to deviations from intended interventions; bias due to missing outcome data; bias in measurement of the outcome; bias in selection of their ported result. A proposed judgment about the risk of bias arising from each domain is generated by an algorithm, based on answers to the signaling questions. Judgment can be “Low”, or “High” risk of bias, or can express “Some concerns”.

Two reviewers (SM and DP) independently judged these domains as having low, moderate, serious, or critical risk of bias, or no information. All discrepancies were resolved by the independent opinion of a third reviewer (PL). A study would be judged as having an overall low risk of bias if all the domains were judged as low risk. A study would be considered as having critical risk of bias if one domain was judged as high risk of bias.

The main results of this systematic review included the serological response after fourth dose vaccine in people with impaired immune system (primary endpoint). Furthermore, the safety and clinical effectiveness of the vaccine was evaluated as a secondary endpoint. The immunogenicity indicators included antibody titers, seroconversion rate, and the response of IgG or other specific antibodies to the receptor-binding domain. Indicators for evaluating safety included local adverse reactions and systemic adverse reactions. Data were reported as mean ± standard deviation or median (range), or number (%).

## Results

**Figure 1:**
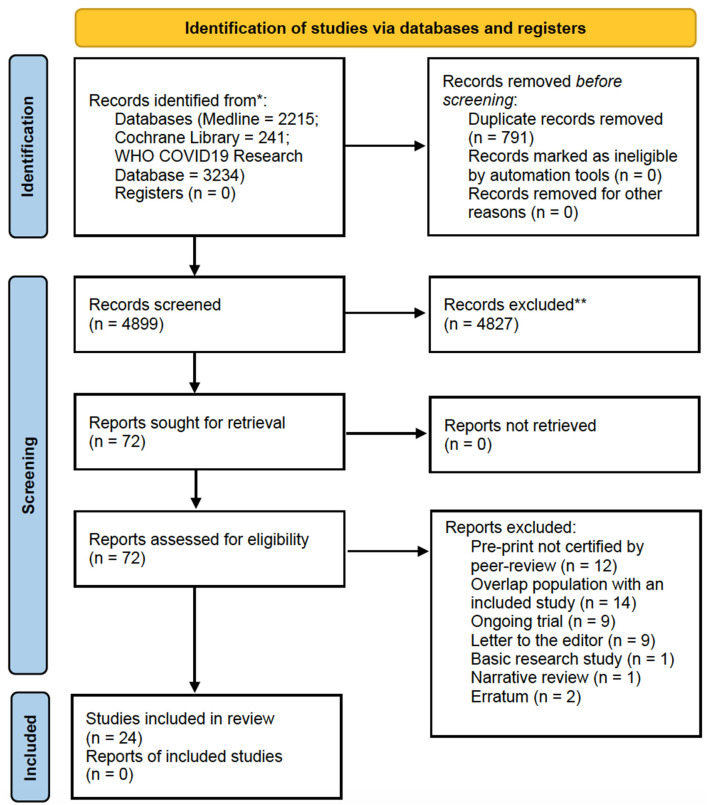
PRISMA 2020 flow diagram for the systematic review.

The selection process of articles and inclusion in the systematic review was summarized in Figure 1, showing the PRISMA flow diagram. The initial search included a total of 5,690 articles. After removing the duplicates, 4,899 articles were screened for keywords and relevance for the title and abstract. The full-text versions of the publications were reviewed in case of uncertainty. Only those that fulfilled the inclusion criteria were included for eligibility assessment. The full-text of these studies were fully examined. A total of 24 articles including 2,838 patients published since January 2023 were comprised in the systematic review (21–44), consisting mainly in retrospective cohort studies, followed by research letters, prospective cohort studies and case series. All the studies involved immunocompromised patients, including solid organ transplant recipients, patients with autoimmune rheumatic disease, patients with HIV and patients with blood cancers or diseases. The majority of studies were carried out in Europe, United States and Israel.

### Risk of bias

By using the Risk of Bias in Non-randomized Studies of Interventions (ROBINS-I), the risk of bias of the studies were summarized in Table 1 (19). In general, the individual studies had a low to moderate range of risk of bias due to adequate approach to the research question and findings, with presence of coherence among the sources of data collection and analysis.

**Table 1:** Methodological quality evaluation of the included non-randomized studies according to ROBINS-1.

**References**	**Bias due to confounding domains relevant to the setting of the study**	**Bias in selection of participants into the study**	**Bias in classification of interventions**	**Bias due to deviations from intended interventions**	**Bias due to missing data**	**Bias in measurement of outcomes**	**Bias in selection of the reported results**
Caillard et al. (21)	PY	PN	PN	PN	PN	N	N
Karaba et al. (22)	PN	N	N	N	PN	PN	N
Kamar et al. (23)	PN	N	N	N	PN	N	N
Teles et al. (24)	PN	PN	N	N	N	N	N
Mitchell et al. (25)	PN	N	PN	N	N	N	N
Osmanodja et al. (26)	PN	N	N	N	PN	PN	N
Aikawa et al. (27)	PN	N	N	N	PN	N	N
Mrake et al. (28)	PN	N	Y	PN	Y	PN	PN
Ntanasis-Stathopoulos et al. (29)	PN	N	PY	PN	PY	PN	PN
Perrier et al. (30)	PN	N	Y	PN	Y	PN	PN
Assawasaksakul et al. (31)	PN	N	Y	PN	Y	PN	PN
Gössi et al. (32)	PN	N	Y	PN	PY	PN	PN
Harberts et al. (33)	PN	N	PY	PN	Y	PN	PN
Benjamini et al. (34)	PN	N	Y	PN	PY	PN	PN
Midtvedt et al. (35)	PN	PN	PY	PN	PY	PN	PN
Peled et al. (36)	PN	N	Y	PN	PY	PN	PN
Thomson et al. (37)	PN	PN	PY	PN	PY	PN	PN
Busà et al. (38)	PN	PN	PY	PN	PY	N	PN
Brandstetter et al. (39)	PN	N	Y	PN	Y	PN	PN
Hod et al. (40)	PN	N	Y	PN	Y	PN	PN
Bjorlykke et al. (41)	PN	N	Y	PN	Y	PN	PN
Lamacchia et al. (42)	PN	PN	PY	PN	PY	N	PN
Affeldt et al. (43)	PN	PN	PY	PN	PY	N	PN
Davidov et al. (44)	PN	N	Y	PN	Y	PN	PN

N, no; PN, probably no; PY, probably yes; Y, yes.

### Main findings

This systematic review reports the use of a fourth dose of vaccine against COVID-19 worldwide in patients with impaired immune system. The characteristics of the included studies are summarized in Table 2, where details of vaccine characteristics and developer information are reported. A total of 2,838 patients were included. Characteristics of included patients are reported in Table 3. The majority of included patients were >50 years old. In twenty studies, 100% of patients were receiving immunosuppressive or immunomodulatory treatment following solid organ transplantation or as a treatment for autoimmune disease or cancer. All studies reported the type of vaccine used. Almost all patients received BNT162b2 or mRNA-1273 as fourth dose (Table 2). The time frame between the third and fourth dose was reported by 19 studies (21–23, 26–31, 33–42), and ranged from 22 to 201 days. All studies but two reported the antibody IgG titer before the 4th dose, using different units of measurement, as reported in Table 4. Table 5 reports the type of antibodies measured, the units of measure and the used assays, which were heterogeneous among the studies. The timing of antibody measurement after the 4th dose was reported by 21 studies and ranged from 14 to 65 days. The values of antibody titers after the 4th dose were reported with different units of measurement by all studies except two. All these studies demonstrated the increase of antibody titers after the fourth dose, both in patients who had a serological strong response and in those who had a weak response after the third dose. One study demonstrated different serological responses according to the evidence of a prior infection with SARS-CoV-2 before the fourth dose (37), reporting higher antibodies levels in patients with history of coronavirus infection. Another study pointed out a weaker serological response in patients who remained seronegative after the third dose (30).

**Table 2:** Design and characteristics of the included studies.

**Author**	**Research type**	**Type of vaccine**	**Country**	**Number of included patients**
Caillard	Retrospective study	BNT162b2 (34 patients)mRNA-1273 (58 patients)	France	92
Karaba	Prospective study	BNT162b2 (10 patients)mRNA-1273 (15 patients)	United States	25
Kamar	Retrospective study	BNT162b2	France	37
Teles	Prospective study	BNT162b2 (11 patients)mRNA-1273 (6 patients)Ad.26.CoV2.S (1 patient)	United States	18
Mitchell	Prospective study	BNT162b2 (46 patients)mRNA-1273 (74 patients)Ad.26.CoV2.S (8 patients)	United States	128
Osmanodja	Retrospective study	mRNA vaccines (217 patients)Others (33 patients)	Germany	250
Aikawa	Prospective study	BNT162b2 (164 patients)	Brazil	164
Mrak	Prospective study	BNT162b2 (29 patients), mRNA-1273 (8 patients)	Austria	37
Ntanasis-Stathopoulos	Prospective study	BNT162b2 (201 patients)	Greece	201
Perrier	Retrospective study	BNT162b2 (507 patients), not specified (16 patients)	France	523
Assawasaksakul	Prospective study	BNT162b2 (28 patients)	Thailand	28
Gössi	Retrospective study	BNT162b2, mRNA-1273	Switzerland	7
Harberts	Prospective study	BNT162b2, mRNA-1273	Germany	36
Benjamini	Prospective study	BNT162b2	Israel	67
Midtvedt	Prospective study	mRNA-1273	Norway	188
Peled	Prospective study	BNT162b2	Israel	90
Thomson	Prospective study	BNT162b2 (239 patients)	United Kingdom	239
Busà	Prospective study	BNT162b2, mRNA-1273	Italy	15
Brandstetter	Retrospective study	BNT162b2 (3 patients), mRNA-1273 (38 patients)	Austria	41
Hod	Prospective study	BNT162b2 (29 patients)	Israel	29
Bjorlykke	Prospective study	BNT162b2, mRNA-1273	Norway	536
Lamacchia	Prospective study	BNT162b2 (8 patients)	Italy	8
Affeldt	Prospective study	BNT162b2, mRNA-1273	Germany	29
Davidov	Retrospective study	BNT162b2 (50 patients)	Israel	50
Total				2,838

**Table 3:** Characteristics of the included patients.

**Author**	**Age, years Median (IQR)**	**Male sex**	**Disease causing impair immune response**	**On medication with immunosuppressive or immunomodulatory therapy (%)**
Caillard	55.9 (47.1–64.2)	64 (69.5%)	Kidney transplant recipients	100%
Karaba	59 (45–66)	11 (44%)	Solid organ transplant recipients	100%
Kamar	NR	NR	Solid organ transplant recipients	NR
Teles	56 (52–66)^**^	5 (27.8%)	Autoimmune rheumatic disease	100%
Mitchell	## 63.5 (54.2–71.6) 62.3 (49.6–69.5) 58.4 (48.4–68)	58 (45.3%)	Solid organ transplant recipients	NR
Osmanodja	61 (51–70)	168 (67%)	Kidney transplant recipients	100%
Aikawa	55.7 (47.3–70.7)	25 (20%)	Autoimmune rheumatic disease	100%
Mrak	62.1 (14.0)^*^	11 (30.6%)	Autoimmune rheumatic disease	100%
Ntanasis-Stathopoulos	67 (15)	114 (56.7%)	Multiple myeloma	100%
Perrier	61.2 (50.9–69.3)#	550(66.7%)	Solid organ transplant recipients	100%
Assawasaksakul	## 39 (11.9)^*^ 53.8 (9.3)^*^	7 (7%) #	Autoimmune rheumatic disease	97%
Gössi	58.5 (17–78)^**^ #	28 (61%)	CAR-T-cell treated patients	100%
Harberts	61.0 (52.5–67.0)	23 (63.9%)	Liver transplant recipients	100%
Benjamini	71.46 (64.90–75.82)	47 (70.1%)	Chronic lymphocytic leukemia	47.8%
Midtvedt	60 ± 12^*^	109 (58%)	Kidney transplant recipients	100%
Peled	57.2 ± 13.8^*^	62 (68.9%)	Heart transplant recipients	100%
Thomson	## 61 (51–68)^**^ 60 (49–67)^**^	149 (62.3%)	Kidney transplant recipients	100%
Busà	58 ± 13^*^	9 (60%)	Solid organ transplant recipients	100%
Brandstetter	## 66.8 (57.45–73.6) 67.75 (56.28–70.83)	26 (63%)	Kidney transplant recipients	100%
Hod	64.2 (54.3–70.4)	16 (55.2%)	Kidney transplant recipients	100%
Bjorlykke	59 (49–67)	229 (43%)	Immune-mediated inflammatory diseases	100%
Lamacchia	58.5 ± 8.9^*^	NR	HIV	100%
Affeldt	55 (20)	22 (61.1%)	Kidney transplant recipients	100%
Davidov	62.7 (53.1–70.6)	26 (52%)	Liver transplant recipient	100%

* mean (standard deviation).

** median (range).

# referred to the entire population of the study.

## data referring to different groups according to the sierological response before the 4th vaccine dose.

NR, not reported; HIV, human immunodeficiency virus.

**Table 4:** Immunological outcomes after 4th dose of vaccination.

**Author**	**Median (IQR) delay between 3rd and 4th dose, days**	**Median (IQR) antibody IgG titer before the 4rd dose, BAU/mL**	**Median (IQR) antibody IgG titer after the 4th dose, BAU/mL**	**Ratio of the median antibody titer after/before the 4th dose**	**Median (IQR) timing of antibody IgG titer exam after the 4th dose, days**
Caillard	68 (61–74.7)	16.4 (5.9–62.3)	145 (27.6–243)	8.8	29 (26–34)
Karaba	93 (28–134)	42.3 (4.9–134.2)	228.9 (115.4–655.8)	5.4	29 (17–38)
Kamar	65 (9)^*^	4 (1–9)^**^α	9.5 (1.7–658)^**^β	2.4	28
Teles	NR	< 0.4–>2,500 γ	1,750 (26–2,500) γ	–	32 (28–34)
Mitchell	NR	207 (11.6–1,500) γ	2,132.5 (96.9–>2,500) γ	10.3	14–28^**^
Osmanodja	64 (55–84)	42% δ	74.2% δ	–	NR
Aikawa	90	29.5 (23.3–37.4) ε	215.8 (180.5–257.9) ε	7.3	30
Mrak	84	0.4 (0.4–8.1)	12.4 (0.4–197.3)	31	30
Ntanasis–Stathopoulos	180 (150–210)^**^	80 ± 3.5% ζ	96.1 ± 3.7% ζ	–	30
Perrier	201 (173–221)	η no = 174 (32.3%); weak = 103 (19.1%); strong = 261 (48.5%)	θ no = 56 (23.8%); weak = 26 (11.1%); strong = 153 (65.1%)	–	31.5^**^
Assawasaksakul	22	88 (49–155)	644 (398–1,041)	7	15
Gössi	NR	< 12 (< 12–>400)^**^ε	30.4 (< 12–400)^**^ε	–	48–59^**^
Harberts	126.0(93.0–148.0)	134.6 ε	1,196.0 ε	9	NR
Benjamini	175 (174–175)	4.3 (0.1–117.65)	41.3 (0.4–1,185)	10	14
Midtvedt	18.0 (9.7–18.3) weeks	4.6 (2.5–32)	1,553 (356–3,703) in 79 patients with >200 BAU/ ml 53 (12–407) in 96 patients sero–negative before dose 4	–	3–4 weeks
Peled	173.4 ± 4.2^*^	12.5 ε	96.9 ε	7.8	16.1 ± 4^*^
Thomson	92–130^**^	3,791 (1,142–5,680) in patients with history of SARS-CoV-2 infection 295 (9.1–1,611) in patients without previous SARS-CoV-2 infection	3,993 (835–5,680) in patients with history of SARS-CoV-2 infection 437 (26–2,211) in patients without previous SARS-CoV-2 infection	–	23–66^**^
Busà	168.3 (116–246)^**^	330.2 (59.02–1,001)	1,020 (366.6–5,486)		65.33 (26–127^**^)
Brandstetter	26 (26–27)	NR	44.7 (17.9–111.6)		26 (26–27)
Hod	175 (164–176)	345 (124–956) 699 (244–2,008) η,	2,118 (761–5,900) 2,489 (1,098–5,640) η		29 (25–33)
Bjorlykke	84	5,087 (1,250–9,081)	6,192 (2,878–11,243)		2–4 weeks
Lamacchia	119 ± 2^*^	NR	NR		7, 30, 60 days
Affeldt	NR	134.4	NR		NR
Davidov	175 (164–176)	345 (124–955)	2,118 (761–5,900)		29 (25–33)

Continuous data are reported as median (IQR range).

BAU, basal antibody units; AU, antibody units; ELU, Elisa laboratory units.

* mean (standard deviation);

** median (range);

*** range.

^α^data referring to 5 patients seropositive before the 4th dose.

^β^data referring to 31 patients seronegative before the 4th dose.

^γ^reported in U/mL.

^δ^serological response rate.

^ε^reported in AU/mL.

^ζ^median neutrilizing antibodies levels.

^η^data referring to different groups according to the sierological response before the 4th vaccine dose.

^θ^235 patients had serological tests after the fourth dose.

NR, not reported.

**Table 5:** Type of antibody measured, units of measure and assays.

**Author**	**Antibodies measured**	**Unit of measure**	**Assay**
Caillard	Anti-spike IgG	WHO BAU/mL	NR
Karaba	Antinucleocapsid antibody (anti-N), anti- RBD Protein, anti-S immunoglobulin IgG ACE2 neutralizing antibodies	WHO BAU/mL	Multiplex chemiluminescent Meso Scale Diagnostics (MSD, Rockville, MD) V-PLEX COVID-19 Respiratory Panel 3 Kit and ACE2 MSD V-PLEX SARS-CoV-2 ACE2 Panel 23 Kit
Kamar	Anti-spike total antibody concentration	WHO BAU/mL	Wantai enzyme-linked immunosorbent assay test
Teles	Anti-RBD Protein Ig	U/mL	Roche Elecsys immunoassay
Mitchell	Anti-RBD Protein Ig Anti-S1 domain of the spike protein	AU/mL	Roche Elecsys immunoassay and EUROIMMUN Anti-SARS-CoV-2 enzyme immunoassay
Osmanodja	Anti-spike protein S1 IgG Anti-RBD Protein Ig	Serological response rate	EUROIMMUN Medizinische Labordiagnostika AG and Roche Elecsys immunoassay
Aikawa	Total IgG against the SARS-CoV-2 S1 and S2 protein Circulating NAb	AU/mL	Chemiluminescent immunoassay on the ETI-MAX-3000, LIAISON SARS-CoV-2 S1/S2 IgG kit, DiaSorin SARS-CoV-2 sVNT kit GenScript
Mrak	Anti-RBD Protein Ig Anti-S1 domain of the spike protein	BAU/ml	Roche Elecsys immunoassay
Ntanasis-Stathopoulos	antibody-mediated reduction of SARS-CoV-2 RBD binding to the human host receptor angiotensin converting enzyme type 2	Serological response rate	cPass SARS-CoV-2 Nabs Detection Kit (GenScript, Piscataway, NJ)
Perrier	Anti-RBD Protein Ig	BAU/ml	Wantai SARS-CoV-2 Ab ELISA (Beijing Wantaï Biological PharmacyEnterprise), VIDAS SARS CoV-2 IgG II ELF Aassay (Biomérieux), Alinityi SARS-CoV-2 IgG II Quantassay (Abbott), Elecsysanti-SARS CoV-2 S assay (Roche Diagnostics) Atellica sCOVG IgG assay (Siemens Healthineers).
Assawasaksakul	Anti-RBD of the SARS-CoV-2 spike protein	AU/ml	SARS-CoV-2IgG II Quant assay (Abbott Diagnostics)
Gössi	Anti-SARS-CoV-2 IgG antibodies binding to S1 and S2 antigens	AU/ml	automated immunoassay analyzer Liaison^®^ XL by DiaSorin, Saluggia, Italy
Harberts	Anti-RBD Protein Ig	AU/ml	Roche Elecsys immunoassay
Benjamini	(IgG), aimed at the SARS-CoV-2 S protein receptor–binding domain (RBD)	BAU/mL	SARS-CoV-2 IgG II Quant (AbbottLaboratories, Abbott Park, Illinois),
Midtvedt	Anti-SARS-CoV-2 (Wuhan) receptor-binding domain (RBD) binding-(x-axis) and neutralizing-antibodies	BAU/mL	NR
Peled	SARS-CoV-2 anti-RBD IgGantibodies; IgG against sublineage B.1 of the wild-type virus, the B.1.617.2(delta) variant and the B.1.1.529(omicron) variant	AU/ml	SARS-CoV-2 IgGIIQuant assay, Abbott, USA and live virus microneutralization assays
Thomson	Antibodies to nucleocapsid protein (anti-NP)	BAU/mL	Abbott Architect SARS-CoV-2 IgG 2 step chemiluminescent immunoassay (CMIA)
Busà	IgG antibodies against S1 and S2 fragments of the Spike protein	BAU/mL	Chemiluminescent immunoassay (CLIA) LIAISON^®^ Trimeric SARS-CoV-2 S1/S2 IgG (DiaSorin S.p.A., Saluggia, VC, Italy)
Brandstetter	Anti-SARS-CoV-2 antibodies directed against the receptor binding domain of the S1 subunit of the spike (S) protein	BAU/mL	SARS-CoV-2 IgG II Quant assay (Abbott Ireland Diagnostics Division, Sligo, Ireland)
Hod	IgG antibodies to the RBD of the SARS-CoV-2 spike protein	AU/ml	SARS-CoV-2 IgGIIQuantassay, Abbott, USA and live virus microneutralization assays
Bjorlykke	IgG antibodies to the RBD of the SARS-CoV-2 spike protein	BAU/mL	In-house bead-based method validated against a micro-neutralization assay at the Department of Immunology at Oslo University Hospital
Lamacchia	Evaluation of the SARS-CoV-2 spike protein antibodies, including the anti-spike-specific (in trimeric form) IgGs, anti-spike RBD-specific IgGs, anti-spike-specific IgMs, anti-nucleoprotein-specific IgGs, and neutralizing antibodies that block the binding of spike protein with the ACE2 receptor	BAU/ml	chemiluminescent immunoassay (CLIA) LIAISON^®^ Trimeric SARS-CoV-2 S1/S2 IgG (DiaSorin S.p.A., Saluggia, VC, Italy), SARS-CoV-2 IgG II Quant (Abbott Rome, Italy), test for neutralizing antibodies that block the binding of spike protein with the ACE2 receptor (Dia.Pro Diagnostic Bioprobes, Milan, Italy)
Affeldt	IgG antibodies to the RBD of the SARS-CoV-2 spike protein, antibodies targeting additional regions of the spike protein, IgG against the S1 region	BAU/ml	Chemiluminescent microparticle immunoassay (CMIA) SARS-CoV-2 IgG II Quant by Abbott on the automated system Alinity I (Abbott, Abbott Park, IL, USA), (CLIA) LIAISON^®^ SARS-CoV-2 TrimericS IgG assay by DiaSorin, Euroimmun anti-SARS-CoV-2-QuantiVac-ELISA (Enzyme-linked Immunosorbent Assay)
Davidov	Serum titers of IgG antibodies against the SARS-CoV-2 spike RBD	BAU/mL	SARS-CoV-2 IgGIIQuant assay, Abbott, USA and live virus microneutralization assays

BAU, basal antibody units; NR, not reported; NA, not assessed; WHO, World Health Organization; ACE, angiotensin-converting enzyme; Ab, antibody; RBD, Receptor Binding Domain; U, unit; AU, antibody units, ELU, Elisa laboratory units; Nab, neutralizing antibodies.

No serious adverse events after the 4th dose have been reported by 13 studies (21, 23, 26–28, 31, 33–35, 39–41, 44) (Table 6). COVID-19 infection after the fourth dose was reported by 10 authors (21, 23, 28, 29, 32, 34, 40–42, 44) and ranged from 0 to 21%. Overall, all the authors recommended the 4th dose of vaccine against COVID-19 in immunocompromised patients, except for Karaba and Thomson et al. (22, 37).

**Table 6:** Clinical outcomes after 4th dose of vaccination.

**Author**	**Side effects**	**COVID-19 infection**	**4th dose recommended (yes/no)**
Caillard	0 (0%)	1 (1%)	Yes
Karaba	NR	NR	No
Kamar	0 (0%)	0 (0%)	Yes
Teles	NR	NR	Yes
Mitchell	NR	NR	Yes
Osmanodja	0 (0%)	NR	Yes
Aikawa	No serious adverse events	NR	Yes
Mrak	No serious adverse events	1 (2.7%)	Yes
Ntanasis-stathopoulos	NR	0 (0%)	Yes
Perrier	NR	NR	Yes
Assawasaksakul	No serious adverse events	NR	Yes
Gössi	NR	1 (14.2%)	Yes
Harberts	No serious adverse events	NR	Yes
Benjamini	No serious adverse events	14 (21%)	Yes
Midtvedt	No serious adverse events	NR	Yes
Peled	NR	NR	Yes
Thomson	NR	NR	No
Busà	NR	NR	Yes
Brandstetter	No serious adverse events	NR	Yes
Hod	No serious adverse events	9	Yes
Bjorlykke	No serious adverse events	35 (7% of 491)	Yes
Lamacchia	NR	2	Yes
Affeldt	NR	NR	Yes/No
Davidov	No serious adverse events	9 (18%)	Yes

NR, not reported.

## Discussion

Given the continuing COVID-19 emergency associated with the risk of the virus undergoing new mutations and in the view of the fact that a clear reduction in vaccine coverage is evident 4 months after the third dose, it is hypothesized that administration of a fourth dose of vaccine may protect against the risk of severe illness and mortality from coronavirus infection. However, specific considerations must be made for immunocompromised patients. At first, efficacy and safety data on booster doses are less because large trials have often excluded patients with cancer, organ transplant recipients, and those with rheumatological disorders although they constitute 3% of the population (45). On the other side, these patients experience more severe and persistent infection and viral shedding (46) and are at increased risk of death (47).

The present systematic review provides relevant evidence about the current role of the fourth dose of vaccine against COVID-19 in immunocompromised people. At first, several considerations emerge on the population of immunocompromised patients who received the fourth dose. The majority of them had history of solid organ transplant, necessitating long-term immunosuppressive therapy to prevent rejection. Only a few data concern patients with hematological cancers or autoimmune rheumatological disease. Few data on HIV/AIDS patients and on patients with primary immunodeficiencies have been published till now.

It is clear that the availability of vaccines and the vaccination guidelines released from the single countries strongly influenced the use of the fourth dose. The inclusion of different ethnics groups better representing the global population may be limited according to the collected data. These findings generate ethical reflections in addition to scientific considerations.

Second, the types of vaccines used for the fourth dose were almost exclusively mRNA vaccines, produced by the two main companies, despite the large number of different types of vaccines available in the market (48). This observation may be explained because they have generally produced better antibody responses and are to some degree better available at least in the developed countries. Data about the other vaccine platforms should be accrued in the future.

The present review showed that the fourth dose was effective in increasing the antibody titer in immunocompromised patients. How the increase of the antibody titers impacted the rate and severity of COVID-19 infections was less clear, as the majority of studies did not follow the patients after the fourth dose to detect clinical infections, and follow-up data are scarcely reported. The COVID-19 infection rate after the fourth dose was reported only by 10 studies, with different follow-up times, and varied from 0% to 21%. Furthermore, the duration of further protection could not be assessed by the review due to the lack of long-term follow-up after the fourth dose at the time that the articles were published.

It should be emphasized that the circulating viral strain(s) are very different from the Wuhan strain, on the basis of which current vaccines were synthesized, and that antibodies elicited by vaccination are unlikely to provide good protection from mutated strains. However, the protection that vaccines provide is not completely dependent on antibodies, but T-cells play a role, particularly in protecting against severe forms of the disease. In addition, T-cells are likely to be less affected by spike protein mutations, given the promiscuity of antigen recognition by the T-cell receptor. The effect of repeated vaccination on the receptors of the adaptive immune system (whether antibody, T-cell receptor, or B-cell receptor) has not yet been extensively evaluated.

However, overall the systematic review demonstrated a very low rate of early major side effects after the fourth dose in immunocompromised patients. No severe adverse events occurred in 13 studies.

Most of the authors of the included studies recommended the use of the fourth dose, while only Karaba et al. and Thomson et al. dissented (22, 37). Karaba et al. argued that additional dosing of the original vaccines in solid organ transplant recipients might not produce valid defense against infection in the form of neutralizing antibodies against the Omicron variant or new variants generated by Omicron (22). These authors recommend additive approaches, such as modulation of immunosuppressive regimens before booster doses of vaccine, vaccines with alternative antigenic sequences, or extensively neutralizing passive immunity products. Thomson and colleagues (37) asserted that repeated vaccinations do not adequately protect all transplant recipients, as there is a spectrum of immune responses in patients in relation to vaccination and infection and it will disadvantage many immunocompromised people if they are managed as a uniform cohort irrespective of underlying disease, treatment or infection status. They recommend developing a more personalized approach, starting with antibody screening to identify the vaccine non-responders who are likely to be the most immune suppressed and at risk of an adverse outcome with infection.

We also highlight a lack of homogeneity of fourth dose use in the included studies. Particularly, the delay between the third and the fourth dose varied from a minimum median value of 22 days to a maximum of 201 days (Table 4). Also timing of antibody dosage after the fourth dose to assess its efficacy on seroconversion varied, as the type of assays (Tables 4, 5).

Globally, even if the data of the literature should be implemented in the future, our results demonstrate that a fourth dose of mRNA vaccine is effective in increasing the antibody titer and associated with a very low rate of side effects in immunocompromised patients, and therefore in our opinion should be proposed to fragile patients (immunocompromised, elderly) (experts' recommendation). Among the international societies, the World Health Organization recommended a fourth dose for immunocompromised patients (49).

### Limitations

This study has several limitations. Firstly, the included studies are mostly observational and small case series. Secondly, the definition of immunocompromised is not universally shared and varied between studies. We therefore specified in the Methods the definition of immunocompromised that we choose to select the articles. Third, we mainly analyzed the seroconversion rate, which is an indication of an immune response to a vaccine, but is not necessarily related to clinical effectiveness. Data are still few on clinical efficacy endpoints such as COVID-19 infection rates in vaccinated immunocompromised populations. It was not possible to perform a meta-analysis for the heterogeneity of the studies and data and because most of the studies lack a comparator. Finally, the definition of seroconversion and the type of immunoassay used were not standardized across the included studies. Even if vaccine type might influence seroconversion rates after COVID-19 vaccination, the studies included in this review predominantly used mRNA vaccines, limiting potential bias.

### Further issues

For long-term observations, there are relevant hesitations associated with the development of the virus and the specificities of new variants. The wide dissemination of new variants internationally suggests continued viral evolution with the emergence of future variants or sublines, as has been noted for some time. It has been shown in the literature that even a repetitive booster vaccination based on the Wuhan isolate has a limited ability to produce a durable humoral immune response toward a remote variant such as Omicron (50). This highlights the urgency of evaluating and adopting second-generation variant of SARS-CoV-2 vaccines. After all, a possible early availability of second-generation vaccines for the current SARS-CoV-2 variants might favor the administration of the aforementioned rather than the use of a fourth dose of first-generation vaccine. Therefore, further research is useful to study the long-term efficacy of vaccines and the influence of dose, seniority, and manufacturing process on protective efficacy (51).

Based on recent WHO guidance, we emphasize that future studies need to address other gaps in the evidence related to the need for additional booster doses, the duration of vaccine efficacy of inactivated, subunit, and viral vector vaccines over time, and according to disease outcome. Further data are required on the magnitude, extent, and duration of humoral and cell-mediated immune responses to variant (49).

## Conclusion

Our findings highlight the importance of the fourth dose of COVID-19 vaccines for immunocompromised patients. Across the included studies, a fourth dose was associated with improved seroconversion and antibody titer levels. In particular, a fourth dose was associated with increasing immunogenicity in organ transplant recipients and patients with hematological cancers, with a very low rate of serious side effects.

Additional data are needed to define the long-term efficacy of the fourth dose and the influence of dose, age, immune disease and manufacturing process on the protective efficacy of different coronavirus variants.

## Data availability statement

The original contributions presented in the study are included in the article/supplementary material, further inquiries can be directed to the corresponding author.

## Author contributions

SM took care of the data synthesis, collection, and analysis. DP provided expert clinical advice on applied methodology, while PL provided expert clinical advice on community health. All authors critically corrected the manuscript for relevant scientific content and updated and approved the final version.
